# Effects of delayed intraventricular TLR7 agonist administration on long-term neurological outcome following asphyxia in the preterm fetal sheep

**DOI:** 10.1038/s41598-020-63770-6

**Published:** 2020-04-23

**Authors:** Kenta H. T. Cho, Nina Zeng, Praju V. Anekal, Bing Xu, Mhoyra Fraser

**Affiliations:** 1 Department of Physiology, The University of Auckland, Auckland, New Zealand; 2 Biomedical Imaging Research Unit, The University of Auckland, Auckland, New Zealand; 3 The Tsinghua-Berkeley Shenzhen Institute, Tsinghua University, Shenzhen, 518000 People’s Republic of China

**Keywords:** Developmental biology, Neuroscience

## Abstract

In the preterm brain, accumulating evidence suggests toll-like receptors (TLRs) are key mediators of the downstream inflammatory pathways triggered by hypoxia-ischemia (HI), which have the potential to exacerbate or ameliorate injury. Recently we demonstrated that central acute administration of the TLR7 agonist Gardiquimod (GDQ) confers neuroprotection in the preterm fetal sheep at 3 days post-asphyxial recovery. However, it is unknown whether GDQ can afford long-term protection. To address this, we examined the long-term effects of GDQ. Briefly, fetal sheep (0.7 gestation) received sham asphyxia or asphyxia induced by umbilical cord occlusion, and were studied for 7 days recovery. Intracerebroventricular (ICV) infusion of GDQ (total dose 3.34 mg) or vehicle was performed from 1–4 hours after asphyxia. GDQ was associated with a robust increase in concentration of tumor necrosis factor-(TNF)-α in the fetal plasma, and interleukin-(IL)-10 in both the fetal plasma and cerebrospinal fluid. GDQ did not significantly change the number of total and immature/mature oligodendrocytes within the periventricular and intragyral white matter. No changes were observed in astroglial and microglial numbers and proliferating cells in both white matter regions. GDQ increased neuronal survival in the CA4 region of the hippocampus, but was associated with exacerbated neuronal injury within the caudate nucleus. In conclusion, our data suggest delayed acute ICV administration of GDQ after severe HI in the developing brain may not support long-term neuroprotection.

## Introduction

Preterm birth is closely associated with long-term neurodevelopmental disability^1^. While survival rates among preterm infants have significantly improved, rates of neonatal morbidity remain high with 50% of extremely preterm infants displaying cognitive and behavioural difficulties^2–4^. The etiology of preterm brain injury is multifactorial^5^, however a key contributing factor is the greater occurrence of acute hypoxic ischemic encephalopathy (HIE) in moderately preterm infants compared to term^6^. Therapeutic mild hypothermia is now standard care for term newborns after moderate to severe HIE^7^. Despite its benefits, mild hypothermia is not recommended for preterm infants <35 weeks gestational age^8,9^. Thus, there is a considerable unmet need for neuroprotective therapies for preterm infants with acute HIE.

Growing evidence suggests that inflammation plays a critical role in the pathogenesis of HIE and immunomodulatory drugs have therapeutic promise^10^. Toll-like receptors (TLRs) are key regulators of the initial inflammatory response to HI, and while implicated in brain injury, they can be neuroprotective via tolerance (pre-conditioning) and immunomodulation^11^. The TLR7 signaling pathway can modulate both pro- and anti-inflammatory responses in central and peripheral immune cells^12–15^, with the potential to reduce inflammatory injury within the CNS^16–18^. We have previously shown that pre-conditioning with the inflammatory mediator lipopolysaccharide (LPS) before an hypoxic-ischemic (HI) insult in the preterm fetal sheep reduced preterm brain injury, in association with upregulation of TLR7 gene expression and both central and peripheral induction of interferon-β (IFN-β)^19^. In an adult mouse model of stroke, pre-insult exposure to the TLR7 agonist Gardiquimod (GDQ) reduced cerebral infarct size^20^. Further, we have reported that intracerebroventricular (ICV) infusion of GDQ following asphyxia in the preterm fetal sheep significantly improved oligodendrocyte survival within the white matter and subcortical neurons after 3 days post-asphyxial recovery^21^. Additionally, neuroprotection was associated with induction of immunosuppressive M2c-like microglia (cluster of differentiation 163; CD163 immunoreactive microglia) within the white matter and significant elevations in the systemic concentration of anti-inflammatory cytokines IFN-β and interleukin-10 (IL-10). However, HI brain injury can evolve over days and even weeks^22,23^. Indeed, evidence from several animal models of perinatal brain injury suggest cell death typically occurs during the secondary phase of injury, and is often followed by a chronic or tertiary phase of injury characterized by progressive cell death and remodeling^24,25^. Thus, the purpose of this study was to evaluate the effect of delayed GDQ administration on cerebral white and grey matter 7 days after an acute HI insult *in utero* in 0.7 gestation preterm fetal sheep. Neural development of the preterm fetal sheep at this gestational age is broadly equivalent to human brain development at 28–32 weeks gestation^26^.

## Methods

### Animals and surgical preparations

All animal experiments were performed according to procedures approved by the Animal Ethics Committee of The University of Auckland. This study complies with the Animal Research: Reporting *In Vivo* Experiments (ARRIVE) guidelines, developed by the National Centre for the Replacement, Refinement & Reduction of Animals in Research (NC3Rs)^27^. Romney–Suffolk cross fetal sheep were instrumented at 98–99 days of gestation (term ~ 147 days gestation). Ewes were anesthetized by an intravenous injection of propofol (5 mg/kg; AstraZeneca Limited, Auckland, New Zealand), and general anesthesia was maintained using 2–3% isoflurane in O_2_. Ewes received 5 ml of Streptocin (250,000 IU/ml procaine penicillin and 250 mg/ml dihydrostreptomycin; Stockguard Labs, Hamilton, New Zealand) intramuscularly for prophylaxis, 30 minutes before the start of surgery. During surgery, maternal fluid balance was maintained with constant saline infusion (250 ml/h), and the depth of anesthesia, maternal heart rate and respiration in the ewes were constantly monitored by trained anesthetic staff.

Using aseptic techniques, maternal paramedian abdominal and uterotomy incisions were performed to exteriorize the head, neck, and forelimbs of the fetus. Polyvinyl catheters were placed into both left and right brachial arteries of the fetus for pre-ductal blood sampling and mean arterial pressure (MAP) measurements. A further catheter was placed into the amniotic sac and secured to the fetal torso to enable monitoring of intra-amniotic pressure as a reference for fetal blood pressure. A further pair of electrodes was placed subcutaneously over the right shoulder and chest at apex level and sewn across the chest to measure fetal electrocardiogram (ECG). Electroencephalogram (EEG) electrodes were placed on the dura over the parasagittal parietal cortex (5 and 10 mm anterior to the bregma and 5 mm lateral), and a reference electrode placed over the occiput. An ICV catheter was placed into the left lateral ventricle (6 mm anterior and 4 mm lateral to bregma) for infusion of GDQ (Invivogen, San Diego, CA, USA). An inflatable silicone occluder (OC16HD, 16 mm, *In Vivo* Metric, Healdsburg, CA, USA) was placed loosely around the umbilical cord for reversible post-surgical umbilical cord occlusions.

On completion of surgical procedures, the fetus was returned to the uterus and any amniotic fluid loss replenished with warm sterile saline. Thereafter, antibiotics (80 mg Gentamicin, Pharmacia and Upjohn, Rydalmere, New South Wales, Australia) was administered into the amniotic sac and the uterus closed. On closure of the maternal laparotomy incision, the surrounding tissue was infiltrated with a local analgesic, 10 ml 0.5% bupivacaine plus adrenaline (AstraZeneca Ltd., Auckland, New Zealand). All electrode leads and polyvinyl catheters were exteriorized through a trocar hole in the maternal flank. A polyvinyl catheter was inserted into the maternal saphenous vein to provide access for post-operative maternal care and euthanasia. All methodology has been described previously^21^.

### Post-operative care

Following surgery, animals were housed together in individual metabolic cages. Rooms were temperature-controlled (16 ± 1 °C, humidity 50 ± 10%) with a 12 hour light/dark cycle. Ewes were provided with water and food *ad libitum*.

A period of at least 4–5 days recovery was allowed before commencement of experiments. Antibiotics were given intravenously to the ewe each day for 3 days; 600 mg Crystapen (benzylpenicillin sodium, Novartis, Auckland, New Zealand) and 80 mg Gentamicin (Pharmacia and Upjohn, Perth, Australia). Fetal and maternal vascular catheters were kept patent by continuous infusion of heparinized saline (20 U/ml at rate of 0.15–0.20 ml/h). Daily fetal arterial blood samples were collected for measurement of pre-ductal pH, blood gas, base excess (Ciba-Corning Diagnostics 845 blood gas analyser and cooximeter, Massachusetts, USA), glucose and lactate values (YSI model 2300, Yellow Springs, Ohio, USA) to assess well-being. All methodology has been described previously^21^.

### Physiological monitoring

Fetal MAP (Novatrans II, MX860; Medex, Hilliard, OH, USA), corrected for maternal movement by subtraction of amniotic fluid pressure, fetal heart rate (FHR) derived from the ECG, EEG and temperature were recorded continuously from 24 hours before occlusion (102–103 days gestation) until post-mortem (110–111 days gestation) as previously described^19,28^. Physiological data is not reported as part of the present study.

### Experimental protocol

On day 103–104 of gestation, animals were randomly assigned to either sham asphyxia (n = 8), asphyxia + vehicle (n = 7) or asphyxia + GDQ (n = 7) groups. Umbilical cord occlusion (UCO) was induced at 07.30 hours by rapid complete inflation of the umbilical cord occluder for 25 minutes or until blood pressure fell below 8 mmHg or there was fetal asystole^29,30^. Sham control animals received no occlusion. This UCO paradigm is associated with diffuse white matter injury and moderate subcortical neuronal loss^21,31^, comparable to that observed in preterm infants^32^.

The TLR7 ligand, GDQ, was infused into the lateral ventricle of fetuses (asphyxia + GDQ group) using a CMA-100 microinjection pump (Carnegie Medicin, AB, Stockholm, Sweden). Fetuses received a primed continuous infusion of 3.34 mg of GDQ (InvivoGen, San Diego, CA, USA, 1.67 mg/ml, equivalent to approximately 1.8 mg/kg of fetal body weight) dissolved in 2 ml of sterile endotoxin-free modified artificial cerebrospinal fluid (aCSF)^33^, at a rate of 11.1 μl/minutes for 3 hours commencing from 1 to 4 hours after the end of occlusion. This dose was based on evidence of neuroprotection in adult stroke models^20^, and has been recently shown to be neuroprotective in our fetal sheep model^21^. The asphyxia + vehicle animals received an infusion of vehicle alone (aCSF) using the same infusion protocol. Fetal arterial blood samples were collected for measurement of pH, blood gases, glucose and lactate values before (baseline, -30 minutes), during (5 and 17 minutes) and following UCO (1, 4, 6, 24, 72, 96, 120, 144 and 168 hours). Seven days after occlusion, ewes and fetuses were killed by an overdose of sodium pentobarbital intravenously to the ewe (9 g Pentobarb 300, Chemstock International, Christchurch, New Zealand). Fetuses were weighed and sexed. Fetal brains were perfusion fixed *in situ* with 500 ml endotoxin-free heparinized saline followed by 1000 ml of 10% phosphate-buffered formalin, pH 7.4. The fetal brain was removed from the skull and post-fixed in the same fixative for approximately 5 days, then divided into 3 main equivalent blocks (A, B, C) and paraffin embedded using a standard histological procedure. Post-mortem examination and gross histological examination verified proper placement of the ICV catheter. Some local tissue damage was apparent due to ICV catheter placement.

### Histopathology and single-labeling immunocytochemistry

Coronal sections (A, B, C) of brains collected at post-mortem were approximately 3–4 mm in thickness. Regions encompassed within each section were: the anterior section (A; striatum and cortex), the middle section (B; thalamus, dorsal horn of the hippocampus and cortex), and the posterior section (C; thalamus, dorsal and ventral horn of the hippocampus and cortex). Sections were processed and paraffin embedded, then subsequently cut at 10 μm thickness using a microtome passing through the mid-striatum and mid thalamus at 26 and 17 mm, respectfully, to the stereotaxic zero as defined in the stereotaxic atlas for fetal sheep^34^.

Oven dried and xylene deparaffinized sections were rehydrated in a decreasing alcohol series (100%, 95%, 70%), then washed with 0.1 mol/l phosphate buffered saline (PBS). Antigen unmasking was performed using citrate buffer (pH 6.0) by pressure-cooking method (2100 Retriever, Aptum Biologics Ltd, Southampton, UK). Endogenous peroxidase was quenched by incubating the sections with 1% H_2_O_2_ in methanol for 30 minutes in darkness. This method was applied for all antibodies, except Olig-2 in which 1% H_2_O_2_ in PBS was used. Blocking was performed with 3% (vol/vol) normal goat serum (NGS, Life Technologies Limited, Auckland, NZ) in PBS (NGS-PBS), for 1 h at room temperature. Washed slides were then incubated with corresponding primary and secondary antibodies overnight in 3% NGS-PBS at 4 °C.

The following primary antibodies were used. Reactive microglia were labeled with rabbit monoclonal anti-ionized calcium binding adapter molecule-1 (Iba-1) antibody (1:200, AB178680, RRID:AB_2755129, Abcam, Cambridge, England, UK). Reactive astrocytes were labeled with rabbit anti-glial fibrillary acidic protein (GFAP) antibody (1:500, AB68428, RRID:AB_1209224, Abcam). Cells undergoing apoptosis were labeled with rabbit polyclonal anti-cleaved caspase-3, which detects endogenous levels of the large fragment (17/19 kDa) of activated caspase-3 resulting from cleavage adjacent to Asp175 (1:200, 9661, RRID:AB_2341188, Cell Signaling Technology Cleaved Caspase-3 (Asp175), Danvers, MA, USA). Immature/mature oligodendrocytes were labeled with mouse monoclonal anti- 2′-3′-cyclic nucleotide 3′-phosphodiesterase (CNPase) antibody (1:200, AB6319, RRID:AB_2082593, Abcam). Rabbit monoclonal anti-Olig-2 (1:200, AB109186, RRID:AB_10861310, Abcam) was used as a marker of all cells in the oligodendrocyte lineage. Neuronal nuclei (NeuN) were labeled with rabbit monoclonal anti-NeuN (1:200, AB177487, RRID:AB_2532109, Abcam) and proliferative cells were labeled with mouse monoclonal anti-Ki-67 (M7240, RRID:AB_2142367, Dako, Sydney, AU).

Unbound antibody was removed by washing in PBS then incubating overnight with goat anti-mouse biotin-conjugated IgG (CNPase and Ki-67; BA-9200, RRID:AB_2336171, Vector laboratories, California, USA) or 1:200 goat anti-rabbit (Olig-2, NeuN, Iba-1, GFAP and cleaved caspase-3; BA-1000, RRID:AB_2313606, Vector laboratories) in 3% NGS-PBS, at 4 °C. Slides were washed three times with PBS then incubated with 1:200 ExtrAvidin (E2885, Sigma-Aldrich, Auckland, NZ) for 2 hours at room temperature. Sections were treated with diaminobenzidine tetrahydrochloride (DAB, Sigma-Aldrich) to visualize immunoreactivity and nucleus counter-staining was performed using 4′,6-diamidino-2-phenylindole (DAPI, 1:10,000, D1306, ThermoFisher, Victoria, AU). Negative controls in the absence of primary antibody were run in parallel. Sections were then washed in PBS and mounted with citifluor (AF1, Citifluor, Hatfield, USA). All methodology has been described previously^21^.

### Immunofluorescence

Brain sections (10 µm) were rehydrated and antigen retrieval performed as described above. Slides were washed with PBS + 0.1% Triton X-100 (PBST) to permeabilize the tissue. Blocking was performed using 10% NGS in PBST for 1 hour at room temperature. For assessment of proliferating oligodendrocytes, slides were incubated overnight with 1:200 mouse anti-Ki-67 (M7240) and 1:200 rabbit anti-Olig-2 (AB109186, Abcam) in 10% NGS-PBST, at 4 °C. To evaluate microglial polarization, tissue sections were incubated overnight with 1:200 rabbit anti-Iba-1 (AB178680, Abcam) and 1:100 mouse monoclonal anti-human CD163 (MCA1853, RRID:AB_2074540, Bio-Rad, CA, USA) in 10% NGS-PBST, at 4 °C. Slides were washed and incubated for 2 hours with corresponding fluorescent-labeled anti-rabbit secondary antibody (1:500, Alexa Fluor 488, Molecular Probes, Life Technologies, Carlsbad, CA, USA) and anti-mouse secondary antibody (1:500, Alexa Fluor 568, Molecular Probes) in 10% NGS-PBST, at room temperature. Nuclei counter-staining was performed using DAPI (1:10,000, D1306, ThermoFisher, Victoria, AU). Negative controls with the primary antibody omitted were run in parallel. Slides were then washed and mounted with citifluor (AF1, Citifluor, Hatfield, USA). All methodology has been described previously^21^.

### Image analysis and quantification

**Figure 1:**
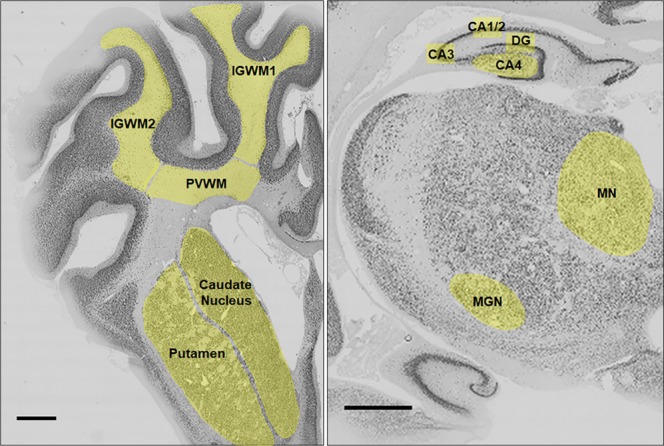
Photomicrographs of coronal sections of a preterm fetal sheep brain representing regions of analysis. Left panel: subcortical periventricular white matter (PVWM) and intragyral white matter (IGWM) tracts, and mid-striatum comprising the caudate nucleus and putamen on sections taken 26 mm anterior to stereotaxic zero. Right panel: thalamic medial nucleus (MN) and medial geniculate nucleus (MGN), dentate gyrus (DG) and cornu ammonis (CA) divisions of the dorsal horn of the anterior hippocampus (CA1/2, CA3, CA4) from sections taken 17 mm anterior to the stereotaxic zero. Scale bar is 2 mm.

**Figure 2:**
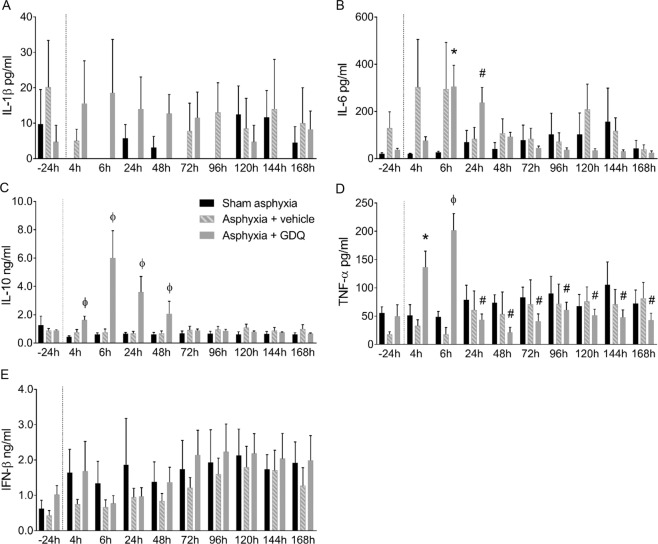
Temporal changes in fetal plasma concentrations of IL-1β (pg/ml), IL-6 (pg/ml), IL-10 (ng/ml), TNF-α (pg/ml) and IFN-β (ng/ml) in sham asphyxia (n = 8), asphyxia + vehicle (n = 7) and asphyxia + GDQ (n = 7) groups in relation to onset of umbilical cord occlusion are represented in A-E, respectively. Dotted line denotes the duration of umbilical cord occlusion. Data presented as mean ± SEM. Statistical significance was determined by split-plot ANOVA followed by Bonferroni’s multiple comparisons test: *p < 0.05 sham asphyxia vs. asphyxia + GDQ; ^#^p < 0.05 asphyxia + vehicle vs. asphyxia + GDQ; ^φ^p < 0.05 sham asphyxia and asphyxia + vehicle vs. asphyxia + GDQ.

Regions of the brain analysed included the subcortical periventricular white matter (PVWM) and intragyral white matter (IGWM) tracts, and the mid-striatum (comprising the caudate nucleus and putamen) on sections taken 26 mm anterior to stereotaxic zero (Fig. 1). The thalamic regions (medial nucleus, MN, and medial geniculate nucleus, MGN), the dentate gyrus (DG) and the cornu ammonis (CA) divisions of the dorsal horn of the anterior hippocampus (CA1/2, CA3, CA4) were evaluated from sections taken 17 mm anterior to the stereotaxic zero. The methodology has been described previously^21^. Whole slide sections were imaged on a Zeiss Axio Imager Z2 microscope with an automated motorized stage (Carl Zeiss AG, Oberkochen, Germany). Serial images were acquired at 10x magnification (0.45 NA, Plan Apochromatic) and collated using the VSlide stitching software (VSViewer, v2.1.117, MetaSystems, Altlussheim, Germany). Regions of interest (ROI) from the whole slide scan were marked by an assessor (K.H.T.C) blinded to the groups by independent coding of slides. Two sections from each animal were counted and averaged. Using ImageJ software (National Institutes of Health, USA, v1.50i), structures of interest were outlined (Fig. 1) and immuno-positive cells were quantified using a macro script customised to individual stains to measure cell density (see Supplementary Figs. S1 & S2). Briefly, for CNPase, GFAP and Iba-1 stains cell bodies were extracted by removing fine cellular processes from background and thresholding with size and circularity filters (sensitivity of automated algorithm versus a human observer (true positive/total actual true): CNPase; 87.4%, GFAP; 89.7%, Iba-1; 89.8%). For cleaved caspase-3, NeuN, Olig-2 and Ki-67 stains, cell nuclei were extracted from background and segmented using size and circularity filters, as well as applying watershed segmentation (sensitivity: cleaved caspase-3; 100%, NeuN; 89.6%, Olig-2; 90.6%, Ki-67; 91.4%). For Olig-2/Ki-67 co-localization, cell nuclei were segmented from individual channels and co-localization was evaluated using the ‘AND’ function (sensitivity: 100%). The full outline of the macro script is provided in Supplementary Fig. 1 and Fig. 2. The range ROI within whole slide scans were 60–150 mm^2^ in the white matter, 70–150 mm^2^ in the striatum (caudate nucleus and putamen), 200–380 μm^2^ within the CA4 hippocampal division, and 25–120 mm^2^ in the thalamic nuclei (MN and MGN). Immunopositivity of NeuN positive cells within the CA1/2, CA3 and DG of the hippocampus displayed high cell density and was quantified by manual assessment of immunostaining at 20x magnification (VSViewer, v2.1.117, MetaSystems). Given the numerous subpopulations of microglia with differing morphological phenotypes and activation states^35,36^, polarization of activated microglia was manually assessed at 20x magnification to quantify the number of ameboid Iba-1 (activated microglia) and CD163 positive cells. Photomicrographs of single-labeling and immunofluorescent sections were taken at 20x and 40x magnifications, respectively (VSViewer, v2.1.117, MetaSystems).

### Cytokine sample collection and analysis

Fetal arterial blood samples were collected for cytokine measurement at baseline (−30 minutes) and following (4, 6, 24, 72, 96, 120, 144, 168 hours) UCO. Cervical puncture was performed at post-mortem for collection of cerebrospinal fluid (CSF). To reduce pre-analytic error, blood samples were immediately placed in pre-chilled tubes with interior coated spray-dried dipotassium ethylenediaminetetraacetic acid (K_2_EDTA, Vacutainer, Becton Dickinson UK Ltd, Plymouth, UK), whereas CSF samples were placed in pre-chilled tubes that did not contain additives. To ensure optimum blood/additive ratio, samples were continuously mixed by gentle immersion, centrifuged at 1,500 g for 10 minutes at 4 °C, and stored at −80 °C. CSF samples were also centrifuged at 1,500 g for 10 minutes at 4 °C and stored at −80 °C. On the day of analysis, samples were thawed on ice, gently vortexed, and briefly centrifuged before dispensing into separate wells of enzyme-linked immunosorbent assay (ELISA) plates. All samples analysed showed no sign of hemolysis.

IFN-β and IL-1β fetal plasma and CSF levels were determined using in-house bovine and ovine ELISA kits, respectively. IFN-β was detected using antibodies specific to the bovine species (Kingfisher Biotech, St. Paul, MN, USA). Standards were bovine recombinant IFN-β (Kingfisher Biotech) and ranged from 0 to 10 ng/ml with a detection sensitivity of 42 pg/ml. IL-1β was detected using antibodies specific to the ovine species (Kingfisher Biotech). Standards were ovine recombinant IL-1β (Kingfisher Biotech) and ranged from 0 to 10 ng/ml with a detection sensitivity of 13 pg/ml. Tumour necrosis factor-α (TNF-α), IL-6 and IL-10 concentrations were measured using in-house ELISAs as previously described^19,37,38^.

### Statistical analysis

A-priori sample size calculation was conducted using the effect size and variance data from our previous study of CNPase and NeuN loss in the PVWM and caudate nucleus, respectively, following asphyxia^21^. Based on analysis of images taken at 20x magnification^21^, our group sizes yielded a statistical power of 80% for both CNPase and NeuN immunoreactivity with an effect size of 2.6 and 1.4, respectively. Fetal biochemical measurements and post-mortem data were evaluated by one-way analysis of variance (ANOVA), followed by Fisher’s least significant difference (LSD) post hoc test when a significant overall effect was found using SPSS v25 (SPSS, Chicago, IL, USA). Cytokine measurements were evaluated by ANOVA, with time as a repeated measure and baseline values as a covariate for split plot analysis using R Statistical Program (v3.3.1 R Statistical Program, The R Foundation for Statistical Computing). Bonferroni post hoc analysis was used to perform pairwise comparisons when a significant overall effect of group or an interaction between group and time was found. Neuropathological data comparisons between groups were performed using ANOVA (v7.03 GraphPad Software, CA, USA). Where significant mean differences were observed between groups and regions, Bonferroni’s post hoc test was used for comparisons. If an effect of region and group was found, the effect of group was assessed for each region separately. All quantitative data are reported as the mean ± standard error of the mean (SEM). The minimum statistical significance threshold was defined as p < 0.05.

## Results

### Umbilical cord occlusion and fetal arterial pressure and heart rate changes

There was no significant difference in the duration of UCO between asphyxia + vehicle and asphyxia + GDQ groups (21.4 ± 0.5 vs. 20.4 ± 0.5 minutes, p > 0.05). In the two occlusion groups, UCO was associated with a significant reduction in both fetal MAP (sham asphyxia; 36.7 ± 1.1 vs. asphyxia + vehicle; 12.6 ± 0.8, and asphyxia + GDQ; 11.7 ± 0.8 mmHg, p < 0.05) and FHR (sham asphyxia; 182.9 ± 6.1 vs. asphyxia + vehicle; 68.7 ± 5.0, and asphyxia + GDQ; 68.7 ± 3.4 bpm, p < 0.05) compared to sham asphyxia, which was not significantly different from each other (p > 0.05).

### Fetal biochemistry

There were no significant differences between groups in pH, blood gases, glucose and lactate in the baseline period (Table 1). UCO was associated with marked hypoxemia and metabolic and respiratory acidosis in both occlusion groups (p < 0.05 vs. sham asphyxia). Post-UCO, most variables normalised within a day. However, in the asphyxia + GDQ group, there were transient changes in pH and PaCO_2_ values between 120–144 hours post-UCO (p < 0.05 vs. sham asphyxia) and a small reduction in lactate between 72–120 hours post-UCO (p < 0.05 vs. asphyxia).

**Table 1:** Fetal blood gases, pH and metabolites during the baseline period, asphyxia, and recovery.

Group	Baseline	5 min UCO	17 min UCO	+1 hour	+4 hour	+6 hour	+24 hour	+72 hour	+96 hour	+120 hour	+144 hour	+168 hour
**pH**												
Sham asphyxia	7.36 ± 0.01	7.36 ± 0.00	7.36 ± 0.00	7.37 ± 0.00	7.37 ± 0.01	7.39 ± 0.01	7.35 ± 0.00	7.35 ± 0.01	7.35 ± 0.00	7.34 ± 0.01	7.35 ± 0.01	7.35 ± 0.01
Asphyxia + vehicle	7.36 ± 0.01	7.01 ± 0.02*	6.81 ± 0.01*	7.30 ± 0.00*	7.36 ± 0.02	7.37 ± 0.02	7.37 ± 0.01	7.36 ± 0.01	7.36 ± 0.01	7.36 ± 0.01	7.35 ± 0.01	7.36 ± 0.01
Asphyxia + GDQ	7.38 ± 0.01	7.00 ± 0.00*	6.82 ± 0.01*	7.32 ± 0.01*	7.39 ± 0.01	7.38 ± 0.01	7.37 ± 0.01	7.37 ± 0.01	7.37 ± 0.01	7.37 ± 0.01*	7.37 ± 0.00*	7.37 ± 0.01
**P**_**a**_**CO**_**2**_ **(mmHg)**												
Sham asphyxia	52.4 ± 1.2	52.6 ± 1.4	51.2 ± 1.5	54.3 ± 1.3	52.7 ± 1.4	53.0 ± 1.0	55.8 ± 1.3	54.4 ± 1.3	51.9 ± 1.6	54.7 ± 1.4	55.3 ± 2.2	53.0 ± 1.8
Asphyxia + vehicle	51.4 ± 1.1	110.0 ± 2.3*	150.7 ± 2.5*	48.9 ± 1.2*	51.9 ± 1.1	51.7 ± 1.7	50.1 ± 1.9*	50.3 ± 1.6	50.5 ± 1.6	52.3 ± 1.6	51.6 ± 1.7	51.3 ± 2.2
Asphyxia + GDQ	50.9 ± 1.2	107.7 ± 3.8*	147.0 ± 2.3*	48.6 ± 1.3*	49.6 ± 0.7	49.9 ± 0.7	49.2 ± 1.5*	47.5 ± 1.5*	47.9 ± 1.4	48.6 ± 1.0*	49.7 ± 0.6*	49.2 ± 1.8
**P**_**a**_**O**_**2**_ **(mmHg)**												
Sham asphyxia	28.9 ± 1.4	27.6 ± 1.0	27.7 ± 1.2	29.0 ± 1.1	28.6 ± 1.7	27.8 ± 1.4	28.8 ± 1.8	29.6 ± 1.3	28.3 ± 2.1	28.4 ± 0.7	27.6 ± 1.0	28.9 ± 0.9
Asphyxia + vehicle	26.8 ± 0.7	4.8 ± 0.2*	5.8 ± 0.4*	31.8 ± 2.6	25.7 ± 1.6	23.2 ± 2.2	29.9 ± 2.0	31.8 ± 1.8	30.7 ± 2.2	28.6 ± 1.8	29.1 ± 1.8	28.7 ± 2.1
Asphyxia + GDQ	28.3 ± 1.1	5.2 ± 0.5*	5.7 ± 0.4*	31.9 ± 1.3	27.3 ± 1.4	27.2 ± 1.1	30.9 ± 1.4	35.0 ± 1.8	33.6 ± 1.4	31.2 ± 1.3	31.0 ± 1.2	29.5 ± 1.3
***Lactate (mmol/L)***												
Sham asphyxia	0.8 ± 0.0	0.7 ± 0.0	0.7 ± 0.0	0.8 ± 0.0	2.5 ± 0.5	1.3 ± 0.2	0.8 ± 0.0	0.8 ± 0.1	0.8 ± 0.1	0.8 ± 0.1	0.8 ± 0.1	0.9 ± 0.1
Asphyxia + vehicle	0.9 ± 0.1	4.2 ± 0.2*	6.8 ± 0.1*	4.4 ± 0.2*	3.8 ± 0.5	3.6 ± 0.8*	1.4 ± 0.2*	1.0 ± 0.1	0.9 ± 0.1	0.9 ± 0.1	0.9 ± 0.1	0.9 ± 0.1
Asphyxia + GDQ	0.8 ± 0.0	4.0 ± 0.1*	6.5 ± 0.3*	4.0 ± 0.1*	3.0 ± 0.6	2.9 ± 0.6	1.1 ± 0.1	0.7 ± 0.0#	0.7 ± 0.0#	0.7 ± 0.0#	0.7 ± 0.0	0.7 ± 0.0
***Glucose (mmol/L)***												
Sham asphyxia	1.3 ± 0.1	1.3 ± 0.1	1.3 ± 0.1	1.4 ± 0.1	1.6 ± 0.1	1.6 ± 0.1	1.2 ± 0.0	1.4 ± 0.1	1.3 ± 0.1	1.3 ± 0.1	1.3 ± 0.1	1.4 ± 0.1
Asphyxia + vehicle	1.3 ± 0.1	0.4 ± 0.0*	0.7 ± 0.1*	1.7 ± 0.1*	1.5 ± 0.1	1.5 ± 0.1	1.5 ± 0.1	1.4 ± 0.1	1.4 ± 0.1	1.2 ± 0.1	1.2 ± 0.1	1.3 ± 0.0
Asphyxia + GDQ	1.3 ± 0.1	0.5 ± 0.1*	0.7 ± 0.2*	1.7 ± 0.1*	1.6 ± 0.1	1.7 ± 0.2	1.5 ± 0.2	1.5 ± 0.1	1.4 ± 0.1	1.3 ± 0.1	1.3 ± 0.1	1.4 ± 0.1

Data presented as mean ± SEM. Abbreviations: UCO, umbilical cord occlusion; PaCO_2_, arterial pressure of carbon dioxide; PaO_2_, arterial pressure of oxygen. Statistical significance was determined by one-way ANOVA followed by Fisher’s LSD post hoc analysis: *p < 0.05 *vs*. sham asphyxia; ^#^p < 0.05 *vs*. asphyxia + vehicle.

### Post-mortem data

There was no significant difference in fetal body weight or fetal sex ratio between experimental groups at post-mortem (Table 2). Brain weight was reduced in both occlusion groups compared with sham asphyxia (30.1 ± 0.1 *vs*. asphyxia + vehicle; 26.7 ± 0.8, and asphyxia + GDQ; 26.3 ± 1.1 g, p < 0.05). Fetal brain per body weight was reduced in both occlusion groups compared with sham asphyxia (17.0 ± 0.5 *vs*. asphyxia + vehicle; 13.4 ± 0.8, and asphyxia + GDQ; 13.9 ± 0.5 g/kg, p < 0.05). There was no significant difference in brain weight or brain per body weight between occlusion groups.

**Table 2:** Post-mortem fetal sex ratio, body and brain weight data.

Group	Sex (M: F)	Fetal body weight (kg)	Brain weight (g)	Brain per body weight (g/kg)
Sham asphyxia	4: 4	1.8 ± 0.1	30.1 ± 0.1	17.0 ± 0.5
Asphyxia + vehicle	3: 4	2.0 ± 0.1	26.7 ± 0.8*	13.4 ± 0.8*
Asphyxia + GDQ	3: 4	1.9 ± 0.1	26.2 ± 0.9*	13.7 ± 0.6*

Data presented as mean ± SEM. Statistical significance was determined by one-way ANOVA followed by Bonferroni’s multiple comparisons test: *p < 0.05 *vs*. sham asphyxia.

### Fetal plasma cytokine concentrations

Baseline concentration of cytokines, IL-1β, IL-6, IL-10, TNF-α and IFN-β, were not significantly different between groups (Fig. 2). Following UCO and throughout the remainder of the experiment, there was no significant difference between groups for IL-1β (Fig. 2A). Post-UCO, the asphyxia + GDQ group was associated with a significant increase in IL-6 concentrations at 6 hours (p < 0.05 *vs*. sham asphyxia) and 24 hours (p < 0.05 *vs*. asphyxia + vehicle) (Fig. 2B). In the asphyxia + GDQ group, IL-10 concentrations were markedly higher from 4 hours until 48 hours post-UCO compared to sham asphyxia and asphyxia + vehicle (p < 0.05) (Fig. 2C). TNF-α concentrations in the asphyxia + GDQ group were also significantly greater at 4 hours (p < 0.05 *vs*. sham asphyxia) and 6 hours post-UCO (p < 0.05 *vs*. sham asphyxia and asphyxia + vehicle), and were significantly lower between 24–72 and 120–168 hours post-UCO (p < 0.03 *vs*. asphyxia + vehicle) (Fig. 2D). Lastly, following UCO and throughout the remainder of the experiment, there was no significant difference in fetal plasma IFN-β concentrations between groups (Fig. 2E).

### Cerebrospinal fluid cytokine concentration

**Figure 3:**
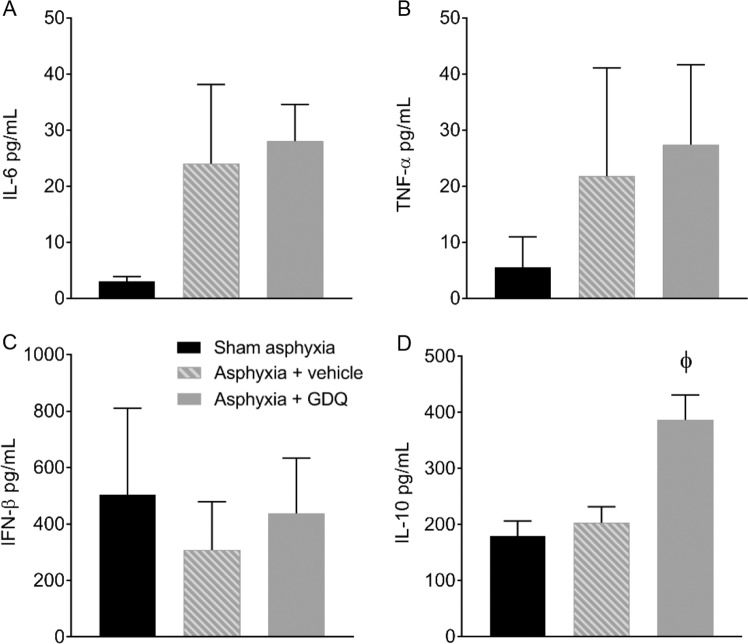
Fetal cerebrospinal fluid (CSF) concentration of IL-6 (pg/ml), TNF-α (pg/ml), IFN-β (pg/ml) and IL-10 (pg/ml) in sham asphyxia (n = 8), asphyxia + vehicle (n = 7) and asphyxia + GDQ (n = 7) groups at 7 days post-occlusion are represented in A-D, respectively. Data presented as mean ± SEM. Statistical significance was determined by one-way ANOVA followed by Bonferroni’s multiple comparisons test: ^ϕ^p < 0.05 sham asphyxia and asphyxia + vehicle vs. asphyxia + GDQ.

Fetal CSF concentrations of the cytokines IL-6, TNF-α and IFN-β were not significantly different between all three groups (Fig. 3A–C). However, IL-10 concentrations in the asphyxia + GDQ group were significantly higher compared to those of sham asphyxia and asphyxia + vehicle (p < 0.05, Fig. 3D). Concentrations of the pro-inflammatory cytokine IL-1β were below the minimum detection value in all groups (data not shown).

### GDQ effects on oligodendrocytes, apoptosis and proliferation

**Figure 4:**
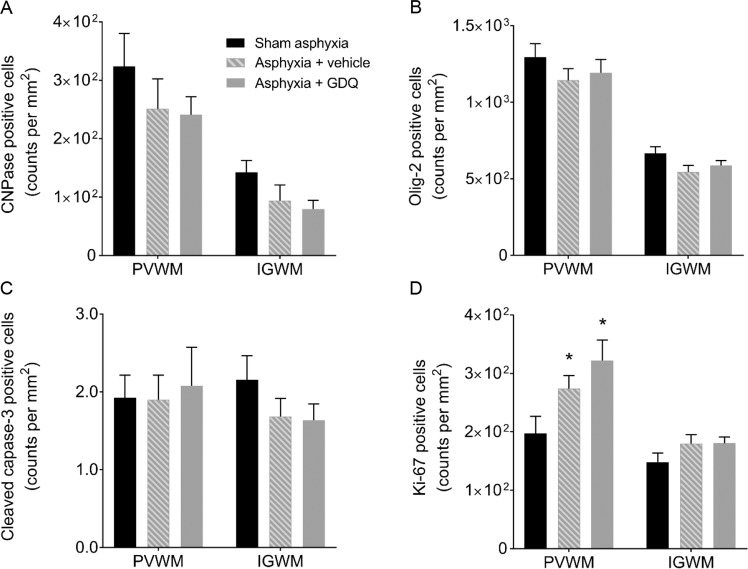
Effect of GDQ on oligodendrocytes, apoptotic and proliferative cells within the periventricular (PVWM) and intragyral white matter (IGWM) regions. Data depict immature and mature oligodendrocytes (CNPase, A), total oligodendrocytes (Olig-2, B), apoptotic cells (cleaved caspase-3, C) and proliferating cells (Ki-67, D) in the PVWM and IGWM of sham asphyxia (n = 8), asphyxia + vehicle (n = 7) and asphyxia + GDQ (n = 7) groups 7 days after umbilical cord occlusion. Statistical significance was determined by two-way ANOVA followed by Bonferroni’s multiple comparisons test: Data presented as mean ± SEM. *p < 0.05 *vs*. sham asphyxia.

**Figure 5:**
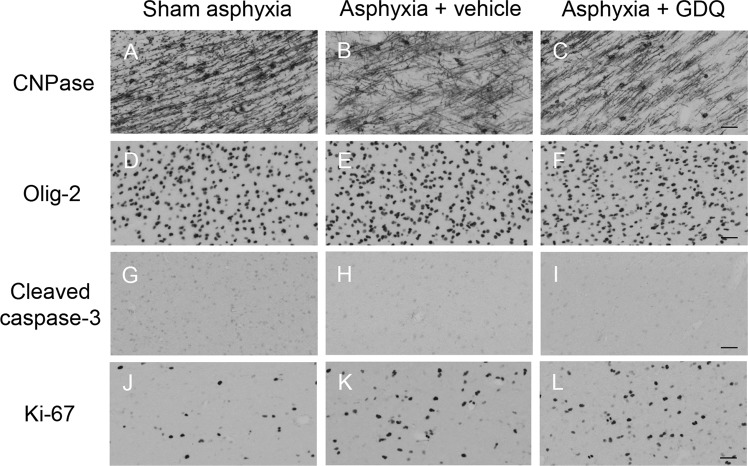
Representative photomicrographs of immature and mature oligodendrocytes (CNPase positive cells, **A**–**C**), total oligodendrocytes (Olig-2 positive cells, **D**–**F**), apoptotic cells (cleaved caspase-3 positive cells, **G**,**H**) and proliferating cells (Ki-67 positive cells, **J**–**L**) in the periventricular white matter (PVWM) from sham asphyxia, asphyxia + vehicle and asphyxia + GDQ groups 7 days after umbilical cord occlusion. Magnification 20×. Scale bar is 50 µm.

To assess whether GDQ treatment affects oligodendrocyte lineage survival, the cell density of immature and mature oligodendrocytes within the PVWM and IGWM were determined using the oligodendroglial cell marker CNPase. Quantitative assessment revealed that in both occlusion groups, there was no significant change in CNPase positive oligodendrocytes compared to sham asphyxia or between occlusion groups (Figs. 4A and 5A–C). Likewise, in both occlusion groups there was no significant change in the proportion of CNPase/Olig-2 positive cells within the PVWM and IGWM compared to sham asphyxia or between occlusion groups (p > 0.05, data not shown). We next performed an assessment of oligodendrocytes throughout their lineage, including mature myelinating oligodendrocytes, using the cell marker Olig-2. As with CNPase, there was no significant difference in Olig-2 cell density in both occlusion groups within the white matter regions compared to sham asphyxia (Figs. 4B and 5D–F). Analysis of cell apoptosis using an antibody that specifically recognizes cleaved caspase-3 (activated caspase-3) revealed no significant change in the number of activated caspase-3 positive cells in the PVWM and IGWM compared to sham asphyxia or between occlusion groups (Figs. 4C and 5G–I).

**Figure 6:**
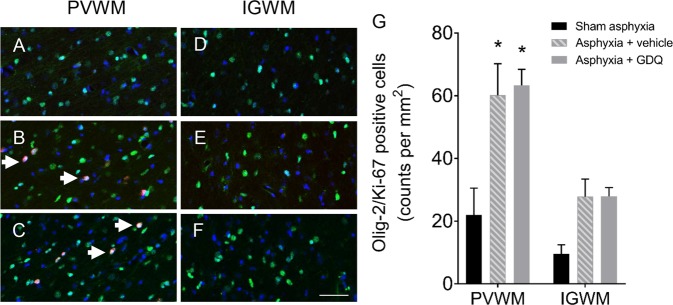
Oligodendrocyte proliferation. Representative photomicrographs of proliferating oligodendrocytes using double immunofluorescent labeling with Ki-67 (red) and Olig-2 (green) within the periventricular (PVWM, **A**–**C**) and intragyral white matter (IGWM, **D**–**F**) from sham asphyxia (**A**,**D**), asphyxia + vehicle (**B**,**E**) and asphyxia + GDQ groups (**C**,**F**) 7 days after umbilical cord occlusion. Dark blue represents DAPI nuclear counterstain. Arrowheads indicate Ki-67/Olig-2 positive staining. Magnification 40×. Scale bar is 50 µm. Data depict the density of Olig-2/Ki-67 positive cells within the PVWM and IGWM in sham asphyxia (n = 8), asphyxia + vehicle (n = 7) and asphyxia + GDQ (n = 7) groups (**G**). Data presented as mean ± SEM. Statistical significance was determined by two-way ANOVA followed by Bonferroni’s multiple comparisons test: *p < 0.05 *vs*. sham asphyxia.

To evaluate compensatory cellular proliferation, single-labeled immunocytochemistry using the proliferative marker Ki-67 was undertaken. Within the PVWM, both occlusion groups revealed a significant increase in proliferating Ki-67 positive cells (p < 0.04 vs. sham asphyxia, Figs. 4D and 5J–L), although there was no significant difference between occlusion groups. On the other hand, there was no significant change in Ki-67 positive cells within the IGWM. To identify proliferating cells, sections were double-labeled with both Ki-67 and Olig-2. As illustrated in Fig. 6, both occlusion groups revealed a significant increase in proliferating oligodendrocytes within the PVWM (p < 0.002 vs. sham asphyxia, Fig. 6A–C,G) compared to sham asphyxia, but there was no significant difference between the occlusion groups. Similar to the Ki-67 labeling on its own, there was no significant change in proliferating oligodendrocytes within the IGWM (Fig. 6D–F,G).

### GDQ effects on density of reactive astrocytes and activated microglia

**Figure 7:**
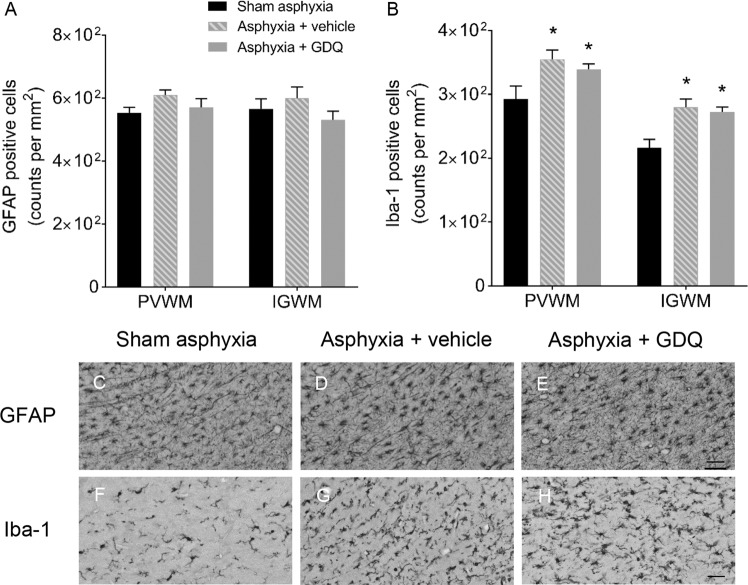
Effects of GDQ on reactive astrogliosis and microglial activation. Data depict the number of astrocytes (GFAP, **A**) and microglia (Iba-1, **B**) in the periventricular white matter (PVWM) and intragyral white matter region (IGWM) in sham asphyxia (n = 8), asphyxia + vehicle (n = 7) and asphyxia + GDQ (n = 7) groups 7 days after umbilical cord occlusion. Data presented as mean ± SEM. Statistical significance was determined by two-way ANOVA followed by Bonferroni’s multiple comparisons test: *p < 0.05 *vs*. sham asphyxia. Representative photomicrographs of astrocytes (GFAP positive cells, **C**–**E**) and microglia (Iba-1 positive cells, **F**–**H**) in the PVWM from sham asphyxia, asphyxia + vehicle and asphyxia + GDQ groups. Magnification 20×. Scale bar is 50 µm.

**Figure 8:**
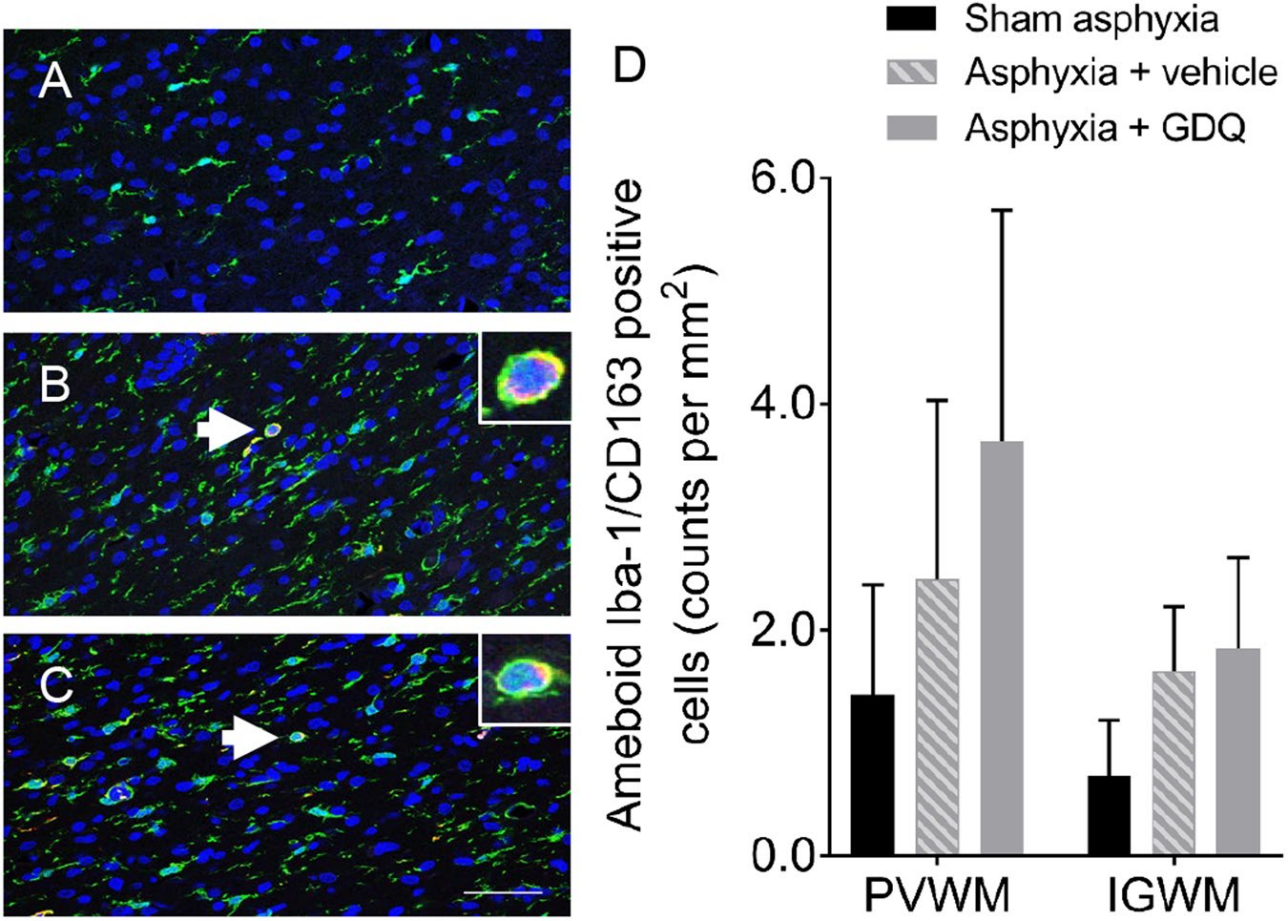
Microglial polarization. Representative photomicrographs of ameboid Iba-1/CD163 positive cells using double-labeling immunofluorescence with Iba-1 (green) and CD163 (red) within the periventricular white matter (PVWM) from sham asphyxia (**A**), asphyxia + vehicle (**B**) and asphyxia + GDQ (**C**) groups 7 days after umbilical cord occlusion. Dark blue represents DAPI nuclear counterstain. Magnification 40×. Scale bar is 50 µm. High magnification images of inserts illustrate ameboid Iba-1/CD163 positive cells. Data depict the effects of GDQ on the density of ameboid Iba-1/CD163 positive cells within the PVWM and intragyral white matter (IGWM) regions in sham asphyxia (n = 8), asphyxia + vehicle (n = 7) and asphyxia + GDQ (n = 7) groups 7 days after umbilical cord occlusion (**D**). Data presented as mean ± SEM. Statistical significance was determined by two-way ANOVA followed by Bonferroni’s multiple comparisons test.

The possible impact of GDQ on reactive astrogliosis and microglial activation was determined. In both occlusion groups there was no significant change in GFAP positive astrocytes within the PVWM and IGWM compared to sham asphyxia (Fig. 7A,C–E). However, the density of microglia (Iba-1 positive cells) within the PVWM and IGWM was significantly increased in both occlusion groups compared to sham asphyxia (p < 0.05), though there was no significant difference between occlusion groups (Fig. 7B,F–H). Double-labeling immunofluorescence revealed the increase in microglia in both occlusion groups did not represent a significant change in the number of ameboid Iba-1 (activated microglia) / CD163 positive cells within the PVWM and IGWM (Fig. 8).

### GDQ effects on neuronal density within hippocampal and selected subcortical regions

**Figure 9:**
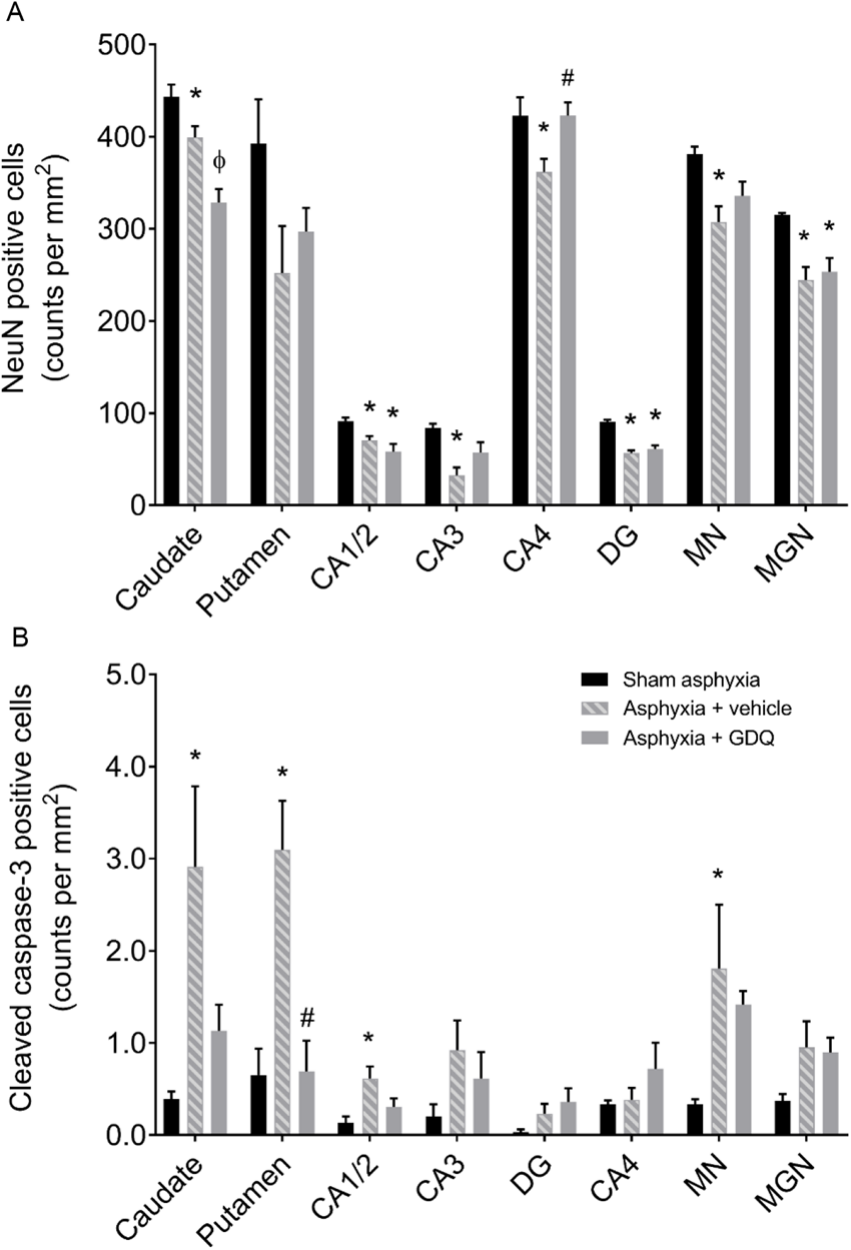
Effects of GDQ on neuronal density and caspse-3 activation within hippocampal and selected subcortical regions. Data depict the effect of GDQ on the density of neurons (NeuN positive cells, **A**) and apoptotic cells (activated caspase-3 positive cells, **B**) within the caudate nucleus, putamen, hippocampal divisions cornu ammonis (CA) 1/2, CA3, CA4 and dentate gyrus (DG), thalamic medial nucleus (MN) and medial geniculate nucleus (MGN) in sham asphyxia (n = 8), asphyxia + vehicle (n = 7) and asphyxia + GDQ (n = 7) groups 7 days after umbilical cord occlusion. Data presented as mean ± SEM. Statistical significance was determined by one-way ANOVA followed by Bonferroni’s multiple comparisons test: *p < 0.05 *vs*. sham asphyxia; ^#^p < 0.05 *vs*. asphyxia + vehicle; ^ϕ^p < 0.05 *vs*. sham asphyxia and asphyxia + vehicle.

**Figure 10:**
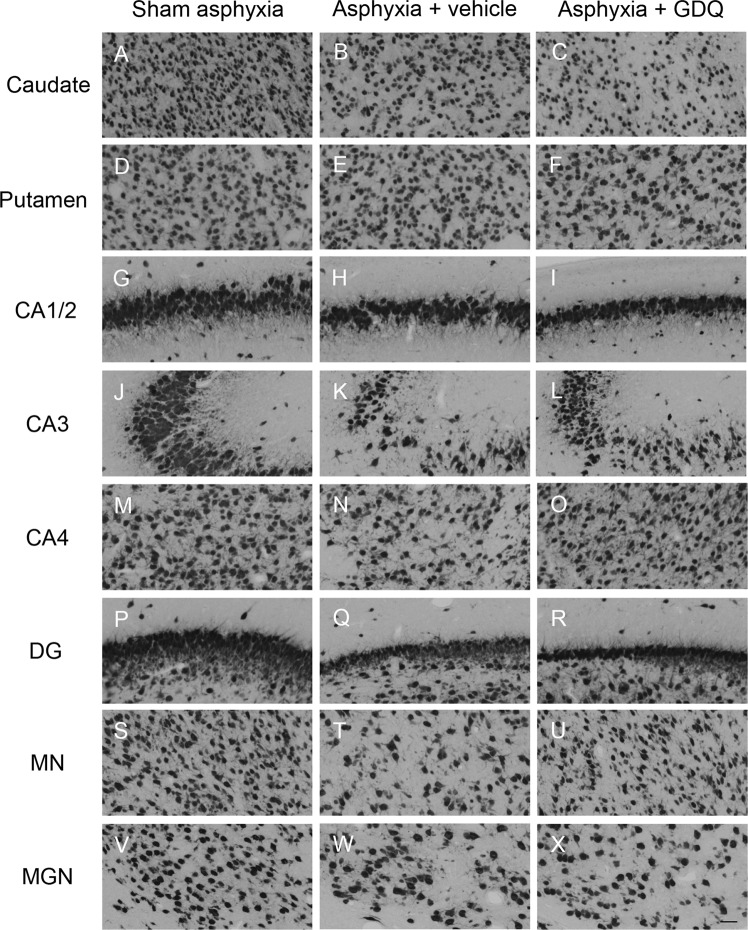
Representative photomicrographs of neurons (NeuN positive cells) in the striatal caudate nucleus (**A**–**C**), putamen (**D**–**F**), hippocampal divisions cornu ammonis (CA) 1/2 (**G**–**I**), CA3 (**J**–**L**), CA4 (**M**–**O**), dentate gyrus DG (**P**–**R**), thalamic medial nucleus (MN, **S**-**U**) and medial geniculate nucleus (MGN, **V**-**X**) in sham asphyxia, asphyxia + vehicle and asphyxia + GDQ groups 7 days after umbilical cord occlusion. Magnification 20×. Scale bar is 50 µm.

The asphyxia + vehicle group exhibited a significant decrease in neuronal density, as visualized by NeuN immunoreactivity, within the striatal caudate nucleus, CA1/2, CA3, CA4, DG of the hippocampus, thalamic MN and MGN compared to sham asphyxia (p < 0.05, Figs. 9A and 10). Similarly, in the asphyxia + GDQ group, among the eight regions assessed, results showed that neuronal density was significantly reduced in the caudate nucleus, CA1/2, DG, and MGN compared to sham asphyxia (p < 0.05). Notably, in the asphyxia + GDQ group there was no significant difference regarding neuronal density in CA3, CA4 and MN regions compared to sham asphyxia. However, when compared to the asphyxia + vehicle group, neuronal density was significantly reduced in the caudate nucleus (p < 0.05), whereas it was significantly improved in the CA4 hippocampal region (p < 0.05).

**Figure 11:**
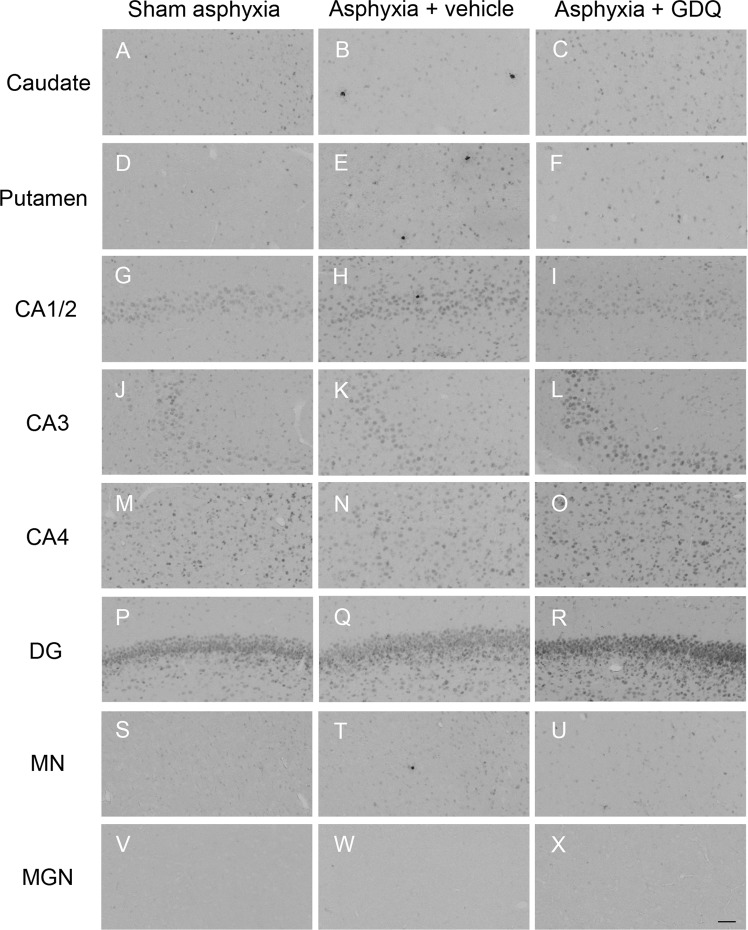
Representative photomicrographs of apoptotic cells (activated caspase-3 positive cells) in the striatal caudate nucleus (**A**–**C**), putamen (**D**–**F**), hippocampal divisions cornu ammonis (CA) 1/2 (**G**–**I**), CA3 (**J**–**L**), CA4 (**M**–**O**), dentate gyrus DG (**P**–**R**), thalamic medial nucleus (MN, **S**–**U**) and medial geniculate nucleus (MGN, **V**–**X**) in sham asphyxia, asphyxia + vehicle and asphyxia + GDQ groups 7 days after umbilical cord occlusion. Magnification 20×. Scale bar is 50 µm.

The asphyxia + vehicle group revealed a significant increase in the number of apoptotic (activated caspase-3 positive) cells within the caudate nucleus, putamen, CA1/2 and MN regions compared to sham asphyxia (p < 0.05, Figs. 9B and 11). In the asphyxia + GDQ group, activated capase-3 cell density was not significantly different between groups in all regions assessed apart from the putamen, which showed a significant reduction in caspase-3 positive cells (p < 0.05 *vs*. asphyxia + vehicle).

## Discussion

The results of this investigation demonstrated that delayed ICV administration of the TLR7 agonist GDQ was not as protective long-term as it was at 3 days recovery following acute severe asphyxia in the preterm fetal sheep^21^. This is reflected by our data indicating GDQ did not significantly change the number of oligodendrocytes in the white matter despite prevailing increases in the concentration of the anti-inflammatory cytokine IL-10 in the CSF and plasma. Furthermore, GDQ administration ameliorated neuronal injury within the hippocampal CA4 sub region, but was without effect in various subcortical regions and increased neuronal loss within the caudate nucleus of the striatum. These data suggest that although GDQ can elicit central and systemic immunomodulation, it may not contribute to long-term neuroprotection, at least using an acute dosing regimen as has been adopted in the present study.

In the current study, we confirm that asphyxia was associated with induction of microglia within the white matter, with no significant difference in astrocytic cell counts, consistent with previous reports in the preterm fetal sheep at 7 days recovery following asphyxia^39,40^. Notably, we found no significant difference in the number of total oligodendrocytes, as indicated by Olig-2 positive cells. These results are in contrast to our previous findings using the same experimental paradigm and dosage regimen, whereby central infusion of the TLR7 agonist GDQ significantly improved survival of not only Olig-2 positive oligodendrocytes, but also CNPase positive oligodendrocytes, 3 days following asphyxia^21^. However, our data is consistent with a previous report speculating net expansion in the pool of oligodendrocytes at 7 days post-asphyxial recovery^28^. Indeed, our finding of robust proliferation of total oligodendrocytes, which includes pre-oligodendrocytes, within the PVWM, as indicated by Olig-2/Ki-67 double-labeling in both asphyxia groups supports this. Nevertheless, while Olig-2 cell density is preserved as a consequence of proliferation of pre-oligodendrocytes following injury, it is associated with failure to terminally differentiate^41,42^. Moreover in support, studies in the preterm fetal sheep report findings of myelin deficits and diffuse loss of immature or mature oligodendrocytes by 7 days post-asphyxial recovery^28,39^. Notwithstanding the above concerns, in the present study we did not observe significant changes to immature or mature oligodendrocyte populations, as indicated by the number of CNPase positive cells. Thus, given these findings a thorough examination of the entirety of the oligodendrocyte lineage, including pre-oligodendrocytes and mature myelinating oligodendrocytes, is required to assess the maturational changes following post-asphyxial treatment as well as asphyxia alone.

Long-term neuronal recovery is also a major end-point of importance. In the present study, asphyxia alone was associated with reduced brain weight and significant neuronal loss in the dorsal striatum caudate nucleus, medial nucleus and medial geniculate nucleus of the thalamus, regions of the dorsal horn of the anterior hippocampus (CA1-4) and dentate gyrus. Such findings agree well with clinical observations of acute asphyxia at birth, whereby moderate to severe damage to subcortical neurons and sparing of the cerebral cortex is detectable by magnetic resonance imaging^43^. Further, neuronal loss was accompanied by on-going upregulation of caspase-3 positive apoptosis in the subcortical nuclei. This pattern of neuronal injury is comparable with previously reported changes in the subcortical grey matter at 7 days post-asphyxial recovery^39^. Presently, despite conferring partial protection within the hippocampus, post-asphyxial administration of GDQ did not significantly improve neuronal injury, but instead further exacerbated neuronal loss within the caudate nucleus of the striatum. This is unlike our previous finding of acute protection against subcortical injury, which exhibited improved survival of neurons within the dentate gyrus and caudate and medial thalamic nucleus at 3 days following asphyxia^21^. Thus, although GDQ confers early, but transient protection of subcortical neurons, it may not be sustainable long-term beyond the secondary phase of recovery after asphyxia.

The underlying reasons for region specific exacerbation of neuronal injury are unknown. Vulnerability of the caudate nucleus to HI injury has been reported in both experimental and clinical studies^44–46^ and likely relates to the susceptibility endowed as a result of neuronal NOS expression in adjacent cells associated with oxidative stress and glutamate receptor activity^47^. The caudate nucleus appears selectively protected by nitric oxide inhibition during asphyxia^48^, since evidence suggests it may assist stabilization of cell function and reduction of excitatory events, such as seizures^49^. Moreover, pro-inflammatory cytokines, such as TNF-α, have been shown to increase the production of nitric oxide in co-cultures of neurons and glial cells^50^ and potentiate glutamate neurotoxicity^51,52^. Given this, it is important to note that analysis of fetal plasma in the present study revealed transient induction of both pro-inflammatory and anti-inflammatory cytokines, such as TNF-α, IL-6 and IL-10, between 4–48 hours recovery. Although the temporal expression of pro-inflammatory and anti-inflammatory cytokines was broadly comparable with our previously demonstrated systemic effects of GDQ^21^ there were dissimilarities in relation to the magnitude of TNF-α induction. Therefore, speculatively, GDQ induced expression of pro-inflammatory cytokines may have contributed, at least in part, to exacerbated neuronal injury.

In our previous 3 day recovery investigation, GDQ was associated with induction of immunosuppressive M2-like microglial cells, as reflected by an increase in the number of CD163/Iba-1 positive cells within the cerebral white matter^21^. However, presently, GDQ-induced M2-like microglial polarization was not sustained by 7 days post-asphyxial recovery. This observation together with the above summation of evidence suggests the possibility that early, but transient, immunomodulatory responses elicited by GDQ may undermine the long-term histological changes. Consistent with this speculation, acute treatment with potential immunomodulators, such as IL-10, IFN-β and minocycline, are only able to exert early transient neuroprotection against ischemic brain injury^53–55^ that is not sustained in the long-term^55–58^. Furthermore, while GDQ was associated with higher CSF concentrations of IL-10 at post-mortem, the magnitude achieved (~400 pg/ml) may have been below the threshold for neuroprotection, since concentrations substantially greater (1 ng/ml) do not attenuate cortical neuronal cell death following oxygen-glucose deprivation *in vitro*^59^. Conversely, there is evidence to support chronic repetitive triggering of TLR7 with periods of treatment-free intervals to be of benefit long-term. For example, in mice repeated administration of R848 (Resiquimod), a synthetic agonist for TLR7, suppressed production of pro-inflammatory cytokines TNF-α and IL-6 and increased production of IL-10^60,61^. Furthermore, using similar dosage regimens, the TLR7 agonist, Imiquimod, reduced experimental autoimmune encephalomyelitis and increased splenic IFN-β production^16^.

Recent studies have demonstrated that TLR7 agonists can induce neuronal cell death dependent on TLR7. Studies by Lehman and co-authors revealed that TLR7 activation in neurons by endogenous ligands, such as miRNA let-7, can induce significant loss of cortical and striatal neurons in adult mice^62^ and this can commence within 3 days after administration, with greater neuronal loss after 2 weeks. Further, *in vitro* studies of cortical neurons derived from mice demonstrate that TLR7-mediated neuronal death occurs after 4 days following stimulation^63^. Thus, the potential of delayed TLR7-mediated cell death may underlie neuronal injury in subcortical regions that exhibit transient protection. Nevertheless, it is important to note that the effect of TLR7 activation on the immature brain may differ from that of the adult brain and following injury. Effects on the normal brain appear different since activation of TLR7 plays a pivotal role in normal neuronal development and function of the brain and that TLR7 can differentially regulate both neuronal and glial cells according to developmental stages^64^. Thus, further studies are required to evaluate the physiological role of TLR7 within the immature brain and determine whether during injury it may account for enhanced susceptibility to neuronal injury.

Some potential limitations of the current histological approach for assessing neural changes require consideration. Pragmatically, histological assessment of brain injury is classically determined by analysis of small regions within key cerebral structures, as well as subjective quantification of various glial and neural cell population by a single assessor^28,39,46,65^. Given the inevitable inconsistencies in area selection across animals, inability to assess non-uniform changes within large structures and potential investigator-to-investigator variability, a robust and unbiased method of analysis of extensive structural regions of the brain is required to confirm prior reports of pathological changes. Therefore, in the current study, we utilized a novel automated cell quantification method to assess white matter regions and subcortical grey matter regions. Our current methodology enabled analysis of larger fields of view of approximately 20-50x within the cerebral white matter. We acknowledge that our current methodology may have overlooked potential changes in CNPase immunoreactivity within specific regions of the white matter. Retrospective power calculations showed that our group size yielded a statistical power of 50% with an effect size of 1.2 for CNPase loss. Given the overall trend for reduced number of CNPase positive cells with asphyxia, the lack of statistical significance may, at least in part, relate to the low statistically power owing to high variability between animals and a relatively small cohort of animals. Thus, a larger cohort may be required to comprehensively report changes in oligodendrocytes, in particular for CNPase positive cells, following asphyxia and treatment. Nevertheless, it is important to appreciate that the current method of analysis may provide better representative regional changes in glial cells and neurons within the brain. An additional limitation of this study relates to our failure to demonstrate a significant increase in the anti-inflammatory cytokine IFN-β in association with GDQ treatment, which is in contrast to our previous findings^21^. Such inconsistencies may relate to different ELISA assay platforms employed in each study.

Finally, whether the adverse effects on striatal neurons is a consequence of modulation of existing injury or effect of treatment alone remain unknown. Future studies to examine the effects of GDQ on the normal brain, the impact of drug dosage and the potential to prolong acute immunomodulatory effects with longer or repeated administration are necessary.

## Conclusion

In conclusion, our findings appear not supportive of a long-term neuroprotective action for delayed ICV administration of the TLR7 agonist, GDQ, following asphyxia in the preterm fetal sheep. This is reflected by our observation that GDQ did not significantly change the number of oligodendrocytes within regions of the white matter vulnerable to injury. Furthermore, whilst GDQ exhibited long-term protective effects within the CA4 region of the hippocampus, it was without effect in various subcortical regions, but did exacerbate neuronal injury within the caudate nucleus. Finally, further studies are required to investigate whether different dosage regimens involving repeated activation of TLR7 may be of benefit long-term.

## Supplementary information

Supplementary information.

Supplementary information 2.

Supplementary information 3.
