# Supplementary material for: Effects of delayed intraventricular TLR7 agonist administration on long-term neurological outcome following asphyxia in the preterm fetal sheep
Source: Sci Rep. 2020 Apr 23;10:6904. doi: 10.1038/s41598-020-63770-6 (PMC7181613; doi:10.1038/s41598-020-63770-6)
Supplement: Supplementary file 1 — Supplementary information. [file 41598_2020_63770_MOESM1_ESM.docx]

**Effects of delayed intraventricular TLR7 agonist administration on long-term neurological outcome following asphyxia in the preterm fetal sheep.**

Kenta H.T. Cho*, Nina Zeng*, Praju V. Anekal^†^, Bing Xu^*‡^ and Mhoyra Fraser***^§^**

**Kenta Hyeon Tae Cho**, *Department of Physiology, The University of Auckland, Auckland, New Zealand. [kenta.cho@auckland.ac.nz](mailto:kenta.cho@auckland.ac.nz).

**Nina Zeng**, *Department of Physiology, The University of Auckland, Auckland, New Zealand.

**Praju Vikas Anekal**, ^†^Biomedical Imaging Research Unit, The University of Auckland, New Zealand. [p.anekal@auckland.ac.nz](mailto:p.anekal@auckland.ac.nz)

**Bing Xu**, *Department of Physiology, The University of Auckland, Auckland, New Zealand and ‡The Tsinghua-Berkeley Shenzhen Institute, Tsinghua University, Shenzhen, Peoples’ Republic of China, 518000. [xu.bing@sz.tsinghua.edu.cn](mailto:xu.bing@sz.tsinghua.edu.cn).

**Mhoyra Fraser * (§corresponding author):** Department of Physiology, The University of Auckland, Auckland 1023, New Zealand. [m.fraser@auckland.ac.nz](mailto:m.fraser@auckland.ac.nz).

**Macro Script Employed to Measure Cell Density**

//Cell counting for single-labelling immunohistochemistry

//KHTC PV 2018

//Initial Variables used. these can be changed later in the macro

AnotherSelection=1;

image_list=newArray("CNP", "GFAP", "IBA", "OLIG2", "NeuN", "Ki67", "Casp3");

DAB="F";

DAPI="A";

name_var_length=1;

file_extn=".tif";

//housekeeping

roiManager("reset"); // these clear the ROIs

roiManager("Centered", "false");

run("Clear Results"); // these clear the results

//user defines image type used.

//the specific list is defined in variables above

Dialog.create("What type of image are you analysing?");

Dialog.addMessage("In addition to Dapi, what type of images are you analysing");

Dialog.addChoice("Image Type:", image_list);

Dialog.show();

Image_Type=Dialog.getChoice();

if (Image_Type=="CNP") {Analysis_Choice=1; }

if (Image_Type=="GFAP") {Analysis_Choice=2; }

if (Image_Type=="IBA") {Analysis_Choice=3; }

if (Image_Type=="OLIG2") {Analysis_Choice=6; }

if (Image_Type=="NeuN") {Analysis_Choice=5; }

if (Image_Type=="Ki67") {Analysis_Choice=6; }

if (Image_Type=="Casp3") {Analysis_Choice=4; }

//ask user to open relevant image and get then to define image type

waitForUser("Open all your images first. Click OK when done");

setBatchMode("hide");

name=getTitle();

tif_index=indexOf(name,file_extn);

core_file_name=substring(name, 0, tif_index-name_var_length);

//replacing image names with generics for processing. the original names are stored in variables

selectWindow(core_file_name+DAB+file_extn); rename("A"); run("Invert");

selectWindow(core_file_name+DAPI+file_extn); rename("B");

//finding white border for removal by cropping. the border is dilated before removal

Dilate_Count=1; Dilate_Itn=5;

selectWindow("A"); run("Duplicate...", " "); rename("mask");

setThreshold(255, 255);

setOption("BlackBackground", true);

run("Convert to Mask");

run("Analyze Particles...", "size=1000-Infinity show=Masks");

run("Invert LUT"); run("Options...", "iterations=Dilate_Itn count=Dilate_Count black do=Dilate");

selectWindow("mask"); close(); selectWindow("Mask of mask"); rename("mask");

run("Invert");

run("Analyze Particles...", "size=1000-Infinity show=Nothing add");

run("ROI Manager...");

run("Merge Channels...", "c1=A c2=B");

selectWindow("RGB");

roiManager("Select", 0);

run("Crop");

roiManager("reset"); // these clear the ROIs

selectWindow("mask"); close();

selectWindow("RGB");

run("Select None");

rename("DAB in Red and Dapi in Green");

setBatchMode("exit and display");

//User selecting ROIs of interest

i2=0;

SelectionName="ROI";

do

{

i2++;

SelectionName="ROI "+i2;

selectWindow("DAB in Red and Dapi in Green");

setTool("freehand");

waitForUser("Select Region. Click OK when done");

roiManager("Add");

roiManager('select', i2-1);

roiManager("rename",SelectionName);

Dialog.create("Do you have another selection?");

Dialog.addMessage("If you have another selection, check Yes \n\n if not leave it Uncheecked");

Dialog.addCheckbox("Yes?", 1) ;

Dialog.show();

AnotherSelection=Dialog.getCheckbox() ;

run("Select None");

}

while (AnotherSelection==1);

//processing and analysing the smaller ROI of interest

for (i3=0; i3<=(i2-1); i3++)

{

Selection_Name="ROI number "+(i3+1);

selectWindow("DAB in Red and Dapi in Green");

roiManager('select', i3);

run("Duplicate...", " ");

rename("X");

run("Split Channels");

selectWindow("X (blue)"); close();

selectWindow("X (red)"); rename("A");

selectWindow("X (green)"); close();

selectWindow("A");

run("Duplicate...", " ");

rename("ROI_Area "+(i3+1));

roiManager("Centered", "true");

roiManager("Select", SelectionName);

run("Multiply...", "value=255");

run("Select None");

setThreshold(254, 255);

run("Convert to Mask");

run("Set Measurements...", "area display redirect=None decimal=2");

run("Analyze Particles...", "size=0-Infinity circularity=0.00-1.00 show=Nothing summarize");

selectWindow("ROI_Area "+(i3+1)); close();

//CNPase analysis

if (Analysis_Choice==1)

{

selectWindow("A");

run("Duplicate...", " ");

run("Median...", "radius=2");

imageCalculator("Subtract create", "A","A-1");

selectWindow("Result of A");

rename("Processes");

setAutoThreshold("Otsu dark");

run("Convert to Mask");

run("Options...", "iterations=2 count=7 black do=Erode");

run("Options...", "iterations=2 count=4 black do=Dilate");

selectWindow("A");

run("Duplicate...", " "); run("Gaussian Blur...", "sigma=1");

rename("A2");

selectWindow("A");

run("Duplicate...", " "); run("Gaussian Blur...", "sigma=10");

rename("A3");

wait(1000);

imageCalculator("Subtract create", "A2","A3");

selectWindow("Result of A2");

rename("A DoG");

imageCalculator("Subtract create", "A DoG","Processes");

selectWindow("Result of A DoG");

run("Minimum...", "radius=1");

setAutoThreshold("Otsu dark");

run("Convert to Mask");

//run("Options...", "iterations=3 count=1 black do=Close");

run("Options...", "iterations=5 count=2 black do=Close");

run("Options...", "iterations=3 count=4 black do=Close");

run("Options...", "iterations=1 count=5 black do=Erode");

run("Watershed");

run("Analyze Particles...", "size=120-5000 circularity=0.45-1.00 show=Masks");

run("Invert LUT");

rename("Cell bodies");

run("Merge Channels...", "c1=[A] c2=[Cell bodies] c3=[A] keep");

run("Add Slice");

selectWindow("Cell bodies");

run("Select All");

run("Copy");

selectWindow("RGB");

run("Paste");

run("Add Slice");

selectWindow("A");

run("Select All");

run("Copy");

selectWindow("RGB");

run("Paste");

rename("RGB "+Selection_Name);

selectWindow("A"); close();

selectWindow("Result of A DoG"); close();

selectWindow("A DoG"); close();

selectWindow("A2"); close();

selectWindow("A3"); close();

selectWindow("A-1"); close();

selectWindow("Processes"); close();

}

//GFAP analysis

if (Analysis_Choice==2)

{

selectWindow("A");

run("Duplicate...", " ");

run("Gaussian Blur...", "sigma=5");

imageCalculator("Subtract create", "A","A-1");

selectWindow("A-1"); close();

selectWindow("Result of A");

run("Minimum...", "radius=0");

setAutoThreshold("Otsu dark");

setOption("BlackBackground", true);

run("Convert to Mask");

run("Options...", "iterations=1 count=5 black do=Close");

run("Analyze Particles...", "size=50-5000 show=Masks");

run("Invert LUT");

run("Options...", "iterations=8 count=2 black do=Close");

selectWindow("Result of A"); close();

selectWindow("Mask of Result of A"); rename("Cell bodies");

run("Merge Channels...", "c1=[A] c2=[Cell bodies] c3=[A] keep");

run("Add Slice");

selectWindow("Cell bodies");

run("Select All");

run("Copy");

selectWindow("RGB");

run("Paste");

run("Add Slice");

selectWindow("A");

run("Select All");

run("Copy");

selectWindow("RGB");

run("Paste");

rename(core_file_name+" "+Selection_Name);

selectWindow("A"); close();

}

//IBA

if (Analysis_Choice==3)

{

selectWindow("A");

run("Duplicate...", " ");

run("Minimum...", "radius=8");

imageCalculator("Subtract create", "A","A-1");

selectWindow("A-1"); close();

selectWindow("Result of A");

run("Minimum...", "radius=0");

setAutoThreshold("Otsu dark");

setOption("BlackBackground", true);

run("Convert to Mask");

run("Options...", "iterations=5 count=3 black do=Close");

run("Options...", "iterations=1 count=1 black do=[Fill Holes]");

run("Analyze Particles...", "size=100-5000 show=Masks");

run("Invert LUT");

run("Options...", "iterations=8 count=2 black do=Close");

selectWindow("Result of A"); close();

selectWindow("Mask of Result of A"); rename("Cell bodies");

run("Merge Channels...", "c1=[A] c2=[Cell bodies] c3=[A] keep");

run("Add Slice");

selectWindow("Cell bodies");

run("Select All");

run("Copy");

selectWindow("RGB");

run("Paste");

run("Add Slice");

selectWindow("A");

run("Select All");

run("Copy");

selectWindow("RGB");

run("Paste");

rename(core_file_name+" "+Selection_Name);

selectWindow("A"); close();

}

//Caspase 3

if (Analysis_Choice==4)

{

selectWindow("A");

run("Duplicate...", " ");

run("Median...", "radius=2");

run("Subtract Background...", "rolling=20 sliding");

setAutoThreshold("Intermodes dark");

//setAutoThreshold("RenyiEntropy dark");

setOption("BlackBackground", true);

run("Convert to Mask");

run("Options...", "iterations=3 count=2 black do=Close");

rename("Cell bodies");

run("Merge Channels...", "c1=[A] c2=[Cell bodies] c3=[A] keep");

run("Add Slice");

selectWindow("Cell bodies");

run("Select All");

run("Copy");

selectWindow("RGB");

run("Paste");

run("Add Slice");

selectWindow("A");

run("Select All");

run("Copy");

selectWindow("RGB");

run("Paste");

rename(core_file_name+" "+Selection_Name);

selectWindow("A"); close();

}

//NeuN

if (Analysis_Choice==5)

{

selectWindow("A");

run("Duplicate...", " ");

run("Median...", "radius=5");

run("Subtract Background...", "rolling=50");

setAutoThreshold("Otsu dark");

setOption("BlackBackground", true);

run("Convert to Mask");

run("Analyze Particles...", "size=100-5000 show=Masks");

run("Invert LUT");

run("Watershed");

selectWindow("Mask of A-1"); rename("Cell bodies");

run("Merge Channels...", "c1=[A] c2=[Cell bodies] c3=[A] keep");

run("Add Slice");

selectWindow("Cell bodies");

run("Select All");

run("Copy");

selectWindow("RGB");

run("Paste");

run("Add Slice");

selectWindow("A");

run("Select All");

run("Copy");

selectWindow("RGB");

run("Paste");

rename(core_file_name+" "+Selection_Name);

selectWindow("A"); close();

selectWindow("A-1"); close();

}

//Ki67

if (Analysis_Choice==6)

{

rename("A");

run("Duplicate...", " ");

run("Minimum...", "radius=8");

imageCalculator("Subtract create", "A","A-1");

selectWindow("Result of A");

setAutoThreshold("Triangle dark");

run("Convert to Mask");

run("Options...", "iterations=4 count=3 black do=Close");

run("Watershed");

run("Analyze Particles...", "size=50-5000 circularity=0.30-1.00 show=Masks");

run("Invert LUT");

rename("Cell bodies");

wait(1000);

run("Merge Channels...", "c1=[A] c2=[Cell bodies] c3=[A] keep");

run("Add Slice");

selectWindow("Cell bodies");

run("Select All");

run("Copy");

selectWindow("RGB");

run("Paste");

run("Add Slice");

selectWindow("A");

run("Select All");

run("Copy");

selectWindow("RGB");

run("Paste");

rename(core_file_name+" "+Selection_Name);

}

selectWindow("Cell bodies");

rename("Number of Cells in "+core_file_name+" "+Selection_Name);

roiManager("Centered", "true");

roiManager("Select", SelectionName);

run("Set Measurements...", "area shape integrated display redirect=None decimal=2");

run("Analyze Particles...", "size=50-5000 circularity=0.20-1.00 show=Nothing summarize add");

selectWindow("Number of Cells in "+core_file_name+" "+Selection_Name); close();

}

setBatchMode("exit and display");

roiManager("Centered", "false");
