# Supplementary material for: Effects of delayed intraventricular TLR7 agonist administration on long-term neurological outcome following asphyxia in the preterm fetal sheep
Source: Sci Rep. 2020 Apr 23;10:6904. doi: 10.1038/s41598-020-63770-6 (PMC7181613; doi:10.1038/s41598-020-63770-6)
Supplement: Supplementary file 2 — Supplementary information 2. [file 41598_2020_63770_MOESM2_ESM.docx]

**Effects of delayed intraventricular TLR7 agonist administration on long-term neurological outcome following asphyxia in the preterm fetal sheep.**

Kenta H.T. Cho*, Nina Zeng*, Praju V. Anekal^†^, Bing Xu^*‡^ and Mhoyra Fraser***^§^**

**Kenta Hyeon Tae Cho**, *Department of Physiology, The University of Auckland, Auckland, New Zealand. [kenta.cho@auckland.ac.nz](mailto:kenta.cho@auckland.ac.nz).

**Nina Zeng**, *Department of Physiology, The University of Auckland, Auckland, New Zealand.

**Praju Vikas Anekal**, ^†^Biomedical Imaging Research Unit, The University of Auckland, New Zealand. [p.anekal@auckland.ac.nz](mailto:p.anekal@auckland.ac.nz)

**Bing Xu**, *Department of Physiology, The University of Auckland, Auckland, New Zealand and ‡The Tsinghua-Berkeley Shenzhen Institute, Tsinghua University, Shenzhen, Peoples’ Republic of China, 518000. [xu.bing@sz.tsinghua.edu.cn](mailto:xu.bing@sz.tsinghua.edu.cn).

**Mhoyra Fraser * (§corresponding author):** Department of Physiology, The University of Auckland, Auckland 1023, New Zealand. [m.fraser@auckland.ac.nz](mailto:m.fraser@auckland.ac.nz).

**Macro Script Employed to Measure Cell Density**

//Cell counting for Olig-2 Ki-67 colocalization

//KHTC PV 2018

//Initial Variables used. these can be changed later in the macro

AnotherSelection=1;

image_list=newArray("Bello", "Hello", "Olig2_Ki67");

DAPI="B";

Olig2="G";

Ki67="O";

name_var_length=1;

file_extn=".tif";

//housekeeping

roiManager("reset"); // these clear the ROIs

roiManager("Centered", "false");

run("Clear Results"); // these clear the results

//user defines image type used.

//the specific list is defined in variables above

Dialog.create("What type of image are you analysing?");

Dialog.addMessage("In addition to Dapi, what type of images are you analysing");

Dialog.addChoice("Image Type:", image_list);

Dialog.show();

Image_Type=Dialog.getChoice();

if (Image_Type=="Olig2_Ki67") {Analysis_Choice=1; }

//ask user to open relevant image and get then to define image type

waitForUser("Open all your images first. Click OK when done");

name=getTitle();

tif_index=indexOf(name,file_extn);

core_file_name=substring(name, 0, tif_index-name_var_length);

//replacing image names with generics for processing. the original names are stored in variables

selectWindow(core_file_name+DAPI+file_extn); rename("A");

selectWindow(core_file_name+Olig2+file_extn); rename("B");

selectWindow(core_file_name+Ki67+file_extn); rename("C");

//finding white border for removal by cropping. the border is dilated before removal

Dilate_Count=1; Dilate_Itn=5;

selectWindow("B"); run("Duplicate...", " "); rename("mask"); run("Invert");

setThreshold(255, 255);

setOption("BlackBackground", true);

run("Convert to Mask");

run("Analyze Particles...", "size=1000-Infinity show=Masks");

run("Invert LUT"); run("Options...", "iterations=Dilate_Itn count=Dilate_Count black do=Dilate");

selectWindow("mask"); close(); selectWindow("Mask of mask"); rename("mask");

run("Invert");

run("Analyze Particles...", "size=1000-Infinity show=Nothing add");

run("ROI Manager...");

run("Merge Channels...", "c1=C c2=B c3=A");

selectWindow("RGB");

roiManager("Select", 0);

run("Crop");

roiManager("reset"); // these clear the ROIs

selectWindow("mask"); close();

selectWindow("RGB");

run("Select None");

rename("I think DAPI in red Olig2 in green Ki67 in blue");

//User selecting ROIs of interest

i2=0;

SelectionName="ROI";

do

{

i2++;

SelectionName="ROI "+i2;

selectWindow("I think DAPI in red Olig2 in green Ki67 in blue");

setTool("freehand");

waitForUser("Select Region. Click OK when done");

roiManager("Add");

roiManager('select', i2-1);

roiManager("rename",SelectionName);

Dialog.create("Do you have another selection?");

Dialog.addMessage("If you have another selection, check Yes \n\n if not leave it Uncheecked");

Dialog.addCheckbox("Yes?", 1) ;

Dialog.show();

AnotherSelection=Dialog.getCheckbox() ;

run("Select None");

}

while (AnotherSelection==1);

//processing and analysing the smaller ROI of interest

for (i3=0; i3<=(i2-1); i3++)

{

Selection_Name="ROI number "+(i3+1);

selectWindow("I think DAPI in red Olig2 in green Ki67 in blue");

roiManager('select', i3);

run("Duplicate...", " ");

rename("X");

selectWindow("X");

run("Duplicate...", " ");

rename("ROI_Area "+(i3+1));

roiManager("Centered", "true");

roiManager("Select", SelectionName);

run("Multiply...", "value=255");

run("8-bit");

setAutoThreshold("Default dark");

//run("Threshold...");

setThreshold(254, 255);

setOption("BlackBackground", true);

run("Convert to Mask");

run("Set Measurements...", "area display redirect=None decimal=2");

run("Analyze Particles...", "size=10000-Infinity circularity=0.00-1.00 show=Nothing summarize");

selectWindow("ROI_Area "+(i3+1)); close();

//Olig2_Ki67 analysis

if (Analysis_Choice==3)

{

run("Split Channels");

selectWindow("X (blue)"); rename("A");

selectWindow("X (green)"); rename("B");

selectWindow("X (red)"); rename("C");

//A is DAPI

selectWindow("A");

run("Subtract Background...", "rolling=50");

run("Median...", "radius=3");

setAutoThreshold("Otsu dark");

//setThreshold(33, 255);

setOption("BlackBackground", true);

run("Convert to Mask");

run("Analyze Particles...", "size=100-Infinity show=Masks");

run("Invert LUT");

run("Watershed");

//B is Olig2

selectWindow("B");

run("Subtract Background...", "rolling=50");

run("Median...", "radius=2");

setAutoThreshold("Triangle dark");

//setThreshold(18, 255);

setOption("BlackBackground", true);

run("Convert to Mask");

run("Analyze Particles...", "size=100-Infinity show=Masks");

run("Invert LUT");

run("Watershed");

//C is Ki67

selectWindow("C");

run("Subtract Background...", "rolling=50");

run("Median...", "radius=3");

setAutoThreshold("Yen dark");

//setThreshold(52, 255);

setOption("BlackBackground", true);

run("Convert to Mask");

run("Analyze Particles...", "size=100-Infinity show=Masks");

run("Invert LUT");

run("Watershed");

imageCalculator("AND create", "Mask of A","Mask of B");

selectWindow("Result of Mask of A");

rename("DAPI and Olig2");

imageCalculator("AND create", "Mask of A","Mask of C");

selectWindow("Result of Mask of A");

rename("DAPI and Ki67");

imageCalculator("AND create", "DAPI and Olig2","DAPI and Ki67");

selectWindow("Result of DAPI and Olig2");

rename("Result of Olig2 and Ki67");

}

wait(1000);

selectWindow("Result of Olig2 and Ki67");

roiManager("Centered", "true");

roiManager("Select", SelectionName);

run("Set Measurements...", "area shape integrated display redirect=None decimal=2");

run("Analyze Particles...", "size=100-Infinity show=Nothing summarize");

roiManager("Centered", "false");

selectWindow("Result of Olig2 and Ki67");
