# Supplementary material for: Effects of delayed intraventricular TLR7 agonist administration on long-term neurological outcome following asphyxia in the preterm fetal sheep
Source: Sci Rep. 2020 Apr 23;10:6904. doi: 10.1038/s41598-020-63770-6 (PMC7181613; doi:10.1038/s41598-020-63770-6)
Supplement: Supplementary file 3 — Supplementary information 3. [file 41598_2020_63770_MOESM3_ESM.docx]

**Supplementary Figures**

**S1**. Macro script for analysis of single labeling immunocytochemistry.

**S2.** Macro script for analysis of Olig-2/Ki-67 co-localization.
